# Effect of Different Skin Penetration Promoters in Halobetasol Propionate Permeation and Retention in Human Skin

**DOI:** 10.3390/ijms18112475

**Published:** 2017-11-21

**Authors:** Paulina Carvajal-Vidal, Mireia Mallandrich, María Luisa García, Ana Cristina Calpena

**Affiliations:** 1 Department of Pharmacy, Pharmaceutical Technology and Physical Chemistry, Faculty of Pharmacy and Food Sciences, University of Barcelona, 08028 Barcelona, Spain; mireia.mallandrich@gmail.com (M.M.); magarlo22@gmail.com or rdcm@ub.edu (M.L.G.); anacalpena@ub.edu (A.C.C.); 2 Institute of Nanoscience and Nanotechnology (IN2UB), University of Barcelona, 08028 Barcelona, Spain

**Keywords:** halobetasol propionate, permeation enhancers, skin permeation, skin inflammation, topical corticosteroid

## Abstract

Halobetasol propionate (HB) is a potent synthetic corticosteroid used against inflammatory skin diseases, such as dermatitis, eczema, and psoriasis, among others. The aim of this study is to define how the presence of different skin penetration enhancers (nonane, menthone, limonene, azone, carene, decanol, linoleic acid and cetiol) affects the penetration and retention in skin of HB. To determine drug penetration through skin, 5% of each promoter was used in an ex vivo system with human skin on Franz cells. The results showed that the highest permeation occurs in the presence of menthone, followed by nonane. Permeation parameters were determined. The in vivo test was assessed, and the formulation containing HB-menthone presented better anti-inflammatory efficacy. These results are useful to generate a specific treatment according to each patient’s needs, and the inflammatory characteristics of the disease.

## 1. Introduction

Skin is considered to be the largest organ of the human body, with a surface area of approximately 2 m^2^. It has an integrated and complex composition that acts as a barrier against exogenous components [1]. This organ is divided into three main layers (starting with the deepest): hypodermis, dermis, and epidermis, the most superficial. The stratum corneum is to the outermost layer of epidermis, which is actually the main barrier to drug permeation [2,3,4]. Despite being a barrier, due to its properties and its structure being rich in capillaries and appendages, skin has been widely studied and used as a route for medication administration [5,6]. Skin can suffer varied alterations or diseases, such as dermatitis, eczema, and psoriasis, among others. A common feature of skin diseases is inflammation, redness, and edema. Although there are various alternative treatments for these symptoms, one of the drug groups of choice is that which is made up of those known as corticosteroids [7,8,9,10].

Corticosteroids are a widely used alternative treatment for inflammatory skin diseases. The drugs in this group can be classified according to their potency or route of administration. The most commonly used topical corticosteroids belong to class I, “superpower corticosteroids”. Among them we find clobetasol, betamethasone, and halobetasol propionate (HB) [11]. Several clinical studies in humans have demonstrated the anti-inflammatory effectiveness of topical corticosteroids against different skin diseases [12,13,14,15]. However, one of the great problems in treatment with topical corticosteroids is that due to their structure and lipophilicity, they can penetrate healthy skin and reach systemic circulation, and may cause toxicity and side effects such as Cushing syndrome, stretch marks, pruritus, acne, hypertension, glaucoma, glycosuria, and growth retardation. Moreover, permeation of corticosteroids may be greater in diseased or altered skin [11,16,17,18,19,20]. In general terms, drugs can permeate through the skin by three routes: the transcellular route, the intracellular route and the trans-appendix route [21]. The route used, and the amount of drug permeated through the skin, depends on the characteristics of the drug and its concentration, the condition of the skin, and external factors, such as occlusion or humidity [19]. In normal conditions, skin can avoid the entrance of external substances or let them pass passively (this is the case with corticosteroids). Due to this, to improve the effect of certain drugs and diminish side effects, it is necessary to modulate their permeability. For this purpose, several alternatives have been described, among which there are the permeation enhancers that can be used in a formulation for improving the transdermal drug delivery by reversibly decreasing the barrier resistance, and at the same time, some of them can be used for enhancing the drug retention in skin [22,23,24,25].

It has been described that these permeation enhancers must have certain properties: they must be well tolerated; they must rapidly begin their action, and they must be suitable for the formulation, the excipients, and for the active ingredient. Enhancers must not cause irreversible damage to the skin and must not have any pharmacological activity [24,26,27]. These enhancers can be classified into various groups depending on their mechanism of action, their structure, and their chemical characteristics. At a general level, some permeation enhancers and their chemical classification are presented in Table 1.

Among the possible mechanisms of action of the permeation enhancers, the following have been described: reversible disruption of the lipid matrix of the skin or on the domains of keratin, alteration in the partitioning coefficient of the drug with the tissue, and alteration of the skin metabolism [24,28]. In both the cosmetic and pharmaceutical industries, the promoters have been widely used and studied to modulate the permeation of various compounds, among them, lactam, terpenes, and alkanols [26,30,31]. Due to the differences between promoter groups, it is necessary to carry out a study to determine which would be the best performing promoter to modulate the penetration through skin, as this depends on the drug. All of the foregoing means that the aim of this study is to define how the presence of different skin penetration enhancers (nonane, menthone, limonene, azone, carene, decanol, linoleic acid, and cetiol) affects the HB penetration and retention in human skin in an ex vivo experiment. Working towards this purpose, we have developed eight different formulations containing the drug and the promoter. We developed and validated a simple, easy-to-use and reliable method for the quantification of HB in ex vivo experimentation using HPLC methodology. Finally, the therapeutic efficacy of the selected formulations was determined in vivo.

## 2. Results

### 2.1. HPLC Validation Methodology

The method was validated for specificity, accuracy, precision, linearity, and sensitivity to analyze HB from samples obtained in ex vivo experimentation on human skin.

**Figure 1:**
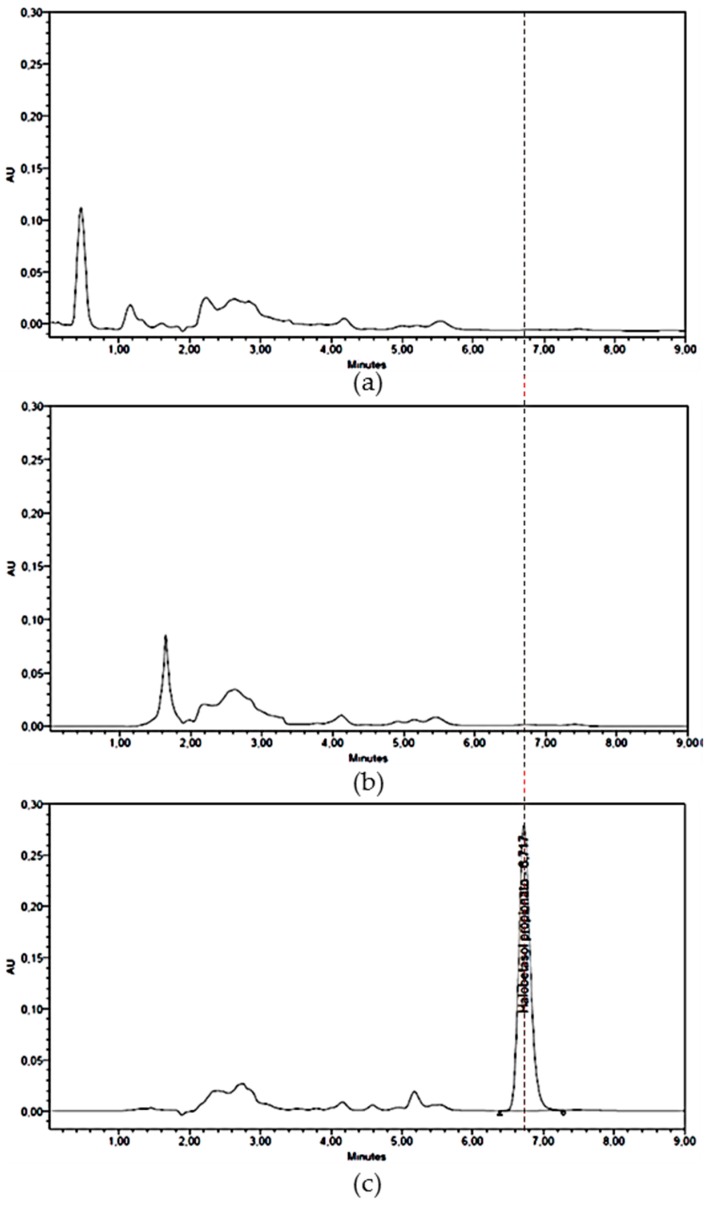

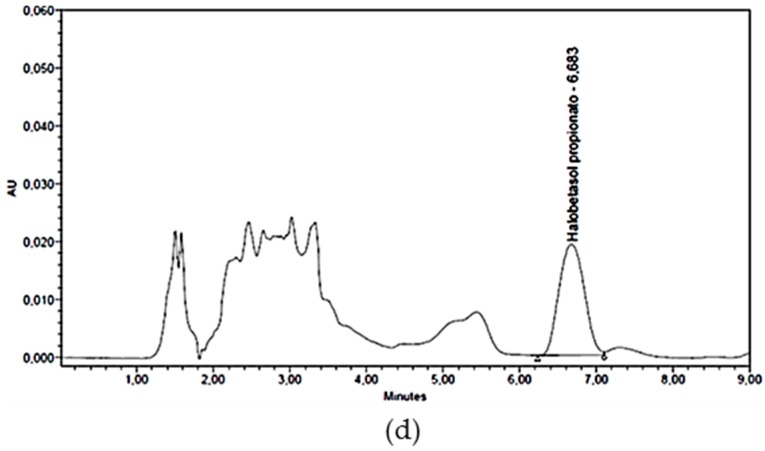
Chromatograms of (**a**) blank of T/w; (**b**) permeated T/w; (**c**) standard 25 μg/mL halobetasol propionate (HB); (**d**) HB permeated in presence of enhancer menthone. (T/w: Transcutol^®^/water).

Under the evaluated conditions described in the methodology, the method is considered specific for the detection and quantification of HB. Figure 1 shows that the chromatograms of the blanks used do not interfere with HB peak. The mean retention time of the drug was 6.6 min.

Accuracy and precision were assessed between the concentrations of 0.5–25 μg/mL. Acquired results are shown in Table 2, expressed as a percentage of relative error (RE) and coefficient of variation (CV). The obtained values for %RE and %CV did not go above 8% and 10% respectively, indicating that the analytical method is accurate and precise in the concentration range under study.

Linearity was established by the measurement of five calibration curves, and ranged between 0.5 and 25 μg/mL. Table 3 shows the areas obtained for each standard concentration of each curve. Statistical analysis ANOVA shows that there are no significant differences between the areas (*p* = 0.6375), indicating that the method is linear in the studied range. From these data, the linear equation is defined by *y* = 116827*x* + 6030, with *r*^2^ = 0.999.

The results of the limit of detection (LOD) and the quantification limit (LOQ) obtained from the lineal equation are 0.34 ± 0.14 and 1.04 ± 0.44, respectively. Considering the results obtained for the parameters of specificity, accuracy, precision, linearity, and sensitivity, the method under validation has proved to be suitable for the analysis and quantification of HB in the range of 0.5–25 μg/mL in ex vivo permeation studies using human skin.

### 2.2. Skin Permeation Assay and Permeation Parameters

**Figure 2:**
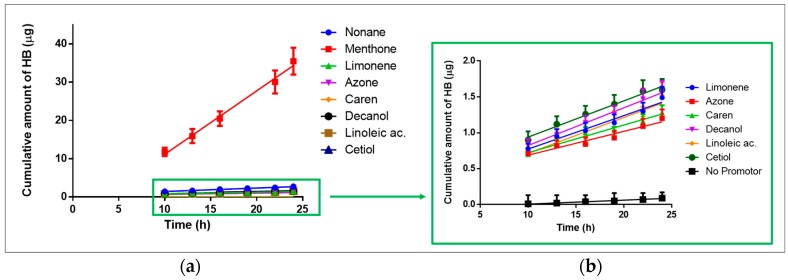
Cumulative permeated amount of HB versus time (h) represented as mean ± SD of six experiments. (**a**) Effect of permeation enhancers in the permeation of HB through skin; (**b**) zoom of the enhancers with lower effect in HB permeation and HB with Transcutol^®^ (No promotor).

HB formulation permeations were performed on human skin for a period of 24 h, following the methodologies described and complying with the guidelines [32,33,34,35]. Transepidermal water loss (TEWL) measurement was performed on all skin pieces, and the values obtained were indicative of a stratum corneum in a condition fit for using in ex vivo permeation tests [36]. Samples were analyzed, and the permeation parameters were determined [37], along with the determination of retained amount of HB per gram of skin. As Transcutol^®^ is a permeation enhancer, a blank solution was used with the same drug concentration, but without any other promoter. For the calculation of the permeation parameters such as flux (J) and permeability coefficient (Kp), a plot of the cumulative amount of drug permeated versus time was made. It can be seen that HB has a better permeation profile in the presence of menthone and nonane compared to the other permeation enhancers studied (Figure 2).

The permeation of HB obtained in the presence of Transcutol^®^ tends to zero in comparison to other enhancers studied. Comparing the drug fluxes in the presence of each promoter, HB permeates 18 times faster in the presence of menthone in comparison to nonane, and on average, 30 times faster than the other enhancers under study, obtaining the lowest flux in the presence of azone (Table 4).

The Kp is calculated from the division of the flux in the initial concentration of the sample (1 mg/mL) so that the differences between the Kp follow the same order as the flux differences. Under 24 h of study, the amount of drug permeated (A_24_) was 35.47 and 2.74 μg for menthone and nonane, respectively. No significant differences were found between the other promoters, and the average permeated amount of drug was 1.45 μg (Table 4). From the results obtained from the extraction of the drug in the skin, we can see that the retention at 24 h of nonane (As = 302.70 μg**·**cm^−2^ g^−1^) is approximately 1.5 times greater than menthone (As = 214.04 μg·cm^−2^ g^−1^). The lowest retention was obtained in the presence of linoleic acid and cetiol (13 times lower than nonane). In spite of finding significant differences in the As of all enhancers (except between linoleic acid and cetiol), the greatest differences are seen when comparing nonane and menthone with all other promoters.

### 2.3. In Vivo Draize Skin Test

The Draize test is used to determine whether or not a substance is an irritant based on the appearance of erythema or edema. As menthone and nonane showed significantly greater effect in enhancing the human skin permeability of HB during ex-vivo experimentation, they were selected for further in-vivo studies. Their in vivo irritant potential effect was assessed in rabbits. For the formulations under study, no edema or erythema formation was found after 24 h of exposure. The individual primary irritancy index determined in three rabbits for each formulation was “0” for both, erythema and edema. Therefore, the formulations HB–menthone and HB–nonane are classified as nonirritant [38].

### 2.4. Efficacy Assay

**Figure 3:**
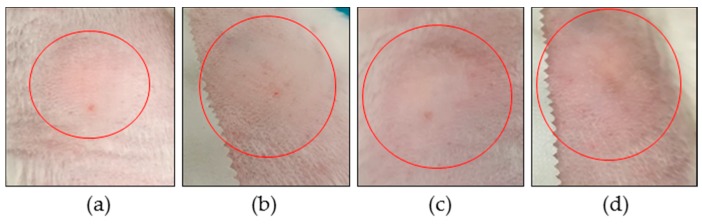
Photographs of histamine wheal suppression test at 30 min. (**a**) HB–menthone; (**b**) HB–nonane; (**c**) HB (in Transcutol^®^); (**d**) control without HB. Red circles indicate wheal formed.

The histamine wheal suppression test is used to determine the anti-inflammatory efficacy of a corticosteroid by forming a bleb when injecting intradermal histamine. It has been described that the maximum effect can be seen between 10 and 30 min post injection [39]. The wheal size was studied in the presence of HB, with and without permeation enhancers. The control corresponds to the bleb formed by histamine in the absence of HB. Figure 3 shows the inflammation produced in the back of the rabbit after 30 min, in which it is observed that the smallest and largest wheal formed corresponds to treatment with HB–menthone and the control, respectively. It also shows a slight redness in the control (d), which is absent in the treated groups (a, b, c), indicating that in the presence of menthone, the response effect is higher.

**Figure 4:**
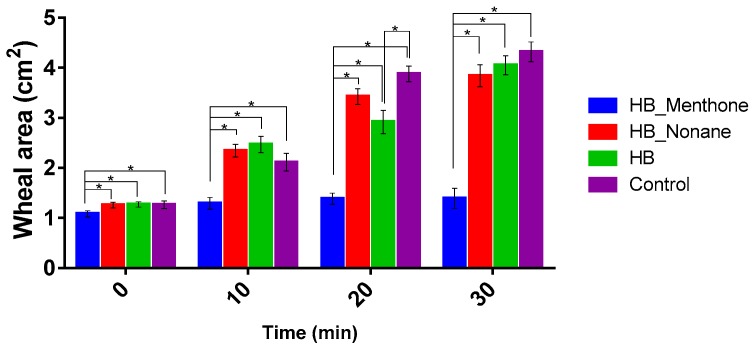
Results of histamine wheal suppression test are represented as mean ± SD. Significant differences are represented with * (*p* < 0.005).

Figure 4 shows the mean of the results. It is evident that in all sampling times, the formulation HB–menthone presents a smaller reaction to the test. This result agrees with ex vivo assays in which menthone was the permeation enhancer with the highest result of HB permeated.

## 3. Discussion

### 3.1. HPLC Validation Methodology

When making a formulation, it is necessary to count on a methodology that allows a reliable quantification of the drug during ex vivo assays. The skin is a complex matrix, and when used during experimentation, its components may interfere with the analytical process, and so may the other components of the formulations under study. This is the reason why a simpler, and easier methodology was developed, with less chemical reagents and at a low cost, in comparison to that provided by pharmacopoeia and other authors for the determination of HB [40]. Numerous methodologies have been described for the detection and quantification of HB in commercial formulations, including high performance liquid chromatography (HPLC), ultra performance liquid chromatography (UPLC) and spectrophotometric techniques [41,42,43,44,45], but so far, no methodology for the quantification of HB samples from ex vivo experimentation with biological tissues has been determined. The HPLC methodology for the determination of HB described by united states pharmacopoeia (USP) [40] uses gradient conditions. It takes approximately one hour to analyze each sample (retention time), and is designed for the quantification of HB without biological matrix, that is to say, without possible biological interferences. In this work we have optimized the methodology described by the USP to perform the analysis of the samples in 7–8 min with an isocratic acetonitrile/water flow which is easier and eight times faster. HB is practically insoluble in water (Cs < 0.007 mg/mL) [46], and most of the techniques described for the determination of non-biological complex samples need pre-treatment, and have to be solubilized in water or a buffer, and this procedure could alter the drug stability [43,45]. Our analytical methods avoid these steps and their related difficulties. Transcutol^®^ is a solubilizer generally used in formulations for skin [47], and delivers a chromatogram with initial peaks that are maintained once it has crossed the barrier. The differences in these initial peaks would be determined by the presence of the promoter used in each formulation, and the presence of some element of the matrix capable of being solubilized with Transcutol^®^.

The validation of a method aims to demonstrate that it is suitable for the purpose it is being used for. Therefore, this analytical method has been validated considering its application and field of use. The parameters of linearity, precision, accuracy, selectivity, and sensitivity have been determined as required by International Conference Harmonization Guidelines (ICH) in the range of 0.5–25 μg/mL, when HB detection is required from samples obtained by ex vivo permeation studies in human skin.

### 3.2. Ex Vivo Studies

Skin is divided into layers, the composition of each one of which varies with the depth at which it is analyzed. The lipid composition of the skin includes sphingolipid, polar, neutral, and apolar lipids among others, which are affected by the promoters when they are used to modulate the permeation of a drug [48]. For a drug to penetrate through the skin, three possible routes are suggested: polar, apolar, or a mixture of both. Although the mechanism of the enhancers’ action is not fully explained, it has been described that they act in altering some of these pathways.

A polar alteration is given by the generation of a conformational change or denaturation of the proteins. An apolar alteration is a consequence of the fluidization of the lipid structure that increases the passive diffusion of a drug [29,49]. Enhancers can also alter the partition coefficient of the drug, *K*_m_ and the permeability of the skin, while maintaining its integrity. Generally, they present at least two simultaneous mechanisms of action [26,29]. Transcutol^®^ is a solvent and drug solubilizer that can act as a permeation promoter with a low toxicity [47]. This solvent was selected due to its ability to solubilize the drug. The results of the permeation profiles indicate that Transcutol^®^ would not act as a permeation accelerator for HB. This result is similar to that found by Bonina & Montenegro [50] in which it was described that for sodium heparin, Transcutol^®^ has no effect as a permeation enhancer. Therefore, Transcutol^®^ was used only for solubilizing the drug and not as a permeation promoter. The permeability enhancers selected for this study have been previously investigated by other authors [1,4,24,26], who obtained varying results regarding the enhancers’ effectiveness, depending on the drug used. They were selected based on their ability to modulate lipophilic compounds. It is known that the action of an enhancer is drug-specific because of the chemical characteristics of both, the enhancer and the drug. Azones have been widely studied: it is believed that their mechanism of action is to act as a solvent of the lipids of the skin, denaturing proteins and modifying the drug diffusion coefficient in the process of permeation through the skin. Several studies have been conducted in which azone increases the permeation of compounds such as antifungal, antimicrobial, triamcinolone, and other corticosteroids, reporting in some cases, irritation problems [26,51,52]. The alkanes exert their promoter effect by being slightly irritating, extensively altering the stratum corneum, and therefore, the barrier function of the epidermis. This increases the permeation of the drugs without damaging the skin [53]. Its activity has been described in drugs such as propranolol hydrochloride [54,55]. On the other hand, fatty acids have been reported to increase drug permeability by causing disturbance of the intercellular lipid bilayer present in the stratum corneum, and that its promoter effect may be influenced by branching and chain length [56]. Fatty acid promoters have been shown to be effective in increasing corticosteroid penetration in betamethasone 17-benzoate [57,58] and hydrocortisone [59].

The highest permeation obtained from HB for all sampling times was in the presence of menthone, a promoter belonging to the terpenes family. The mechanism of action of this promoter has not been fully studied, but it is believed to cause a reversible alteration of skin lipids, thus helping the permeation of anti-inflammatory drugs with a cholesterol-like base structure [31,49,60]. Limonene and carene are hydrocarbon terpenoids, whereas menthone is a ketone terpenoid [61]. This difference could explain the fact that although all three are terpenes, menthone exerts a higher permeating effect. The same may occur with alkanes, on which nonane has a better permeating effect than decanol, due to the differences in the chain length. Because of this, there is the possibility that despite belonging to the same chemical group, not all enhancers produce the same result with a particular drug. When modulating the permeation of drugs such as corticosteroids, it is necessary to consider the possible systemic effect that they have, so that adverse effects can be ruled out, or lessened. The promoters that increased the permeation of HB in descending order were: menthone, nonane, cetiol, decanol, limonene, linoleic acid, careen, and azone. The highest HB permeated amount was 35.47 μg, and the lowest was 1.20 μg at 24 h. The enhancers that allow major retention of HB in skin after 24 h, in descending order, were nonane (302.70 μg·g^−1^ cm^−2^), menthone, decanol, azone, limonene, carene, linoleic acid, and cetiol (22.04 μg·g^−1^ cm^−2^).

### 3.3. In Vivo Studies

The skin tolerance and efficacy tests were performed on rabbits. Although it has been described that their skin may be more permeable to certain compounds, they are animal models which are easy to handle, inexpensive, and they have human-like skin characteristics, all of which make them a good approximation prior to the human determinations [62,63,64]. The Draize test was negative for erythema and edema after 24 h of exposure to HB formulations with menthone and nonane. The drug, Transcutol^®^, and the promoters used, are approved by the food and drug administration (FDA) and can be used in commercial formulations [10], so it was expected that they would not cause any skin alterations in the proportions used.

Several methodologies have been described to determine the efficacy and penetration of topical corticosteroids. Among them, we have the vasoconstriction test, radiolabeling, and micro dialysis [65,66,67]. The histamine-induced wheal suppression test performed has the advantage of being a simple, reliable, reproducible, and non-invasive method compared to other techniques. This test can also mimic the inflammatory conditions such as redness and temperature where a topical corticosteroid would be used. It can be performed using fewer animals per experiment, and allows the study of more than one substance in the same individual, if necessary [39]. After the ex vivo study, it was decided to choose the two formulations with higher permeation profiles in 24 h. HB–menthone and HB–nonane were selected for their in vivo efficacy test. The anti-inflammatory effect was higher for HB–menthone, followed by HB–nonane, both with better effect than the control. These results agree with those obtained in the ex vivo studies, in which HB–menthone exhibited a higher level of permeated HB than HB–nonane, as well as a higher flux. The developed HB–promoter formulations possess an euthermic pH, a characteristic odor, and a clear color, with a pleasant feel on touch. While they have not been tested in humans, they can be predicted to be safe. The maximum recommended dose of HB in commercial formulations should not exceed 50 g/week of a 0.05% HB cream or lotion with a maximum of two weeks treatment period. This corresponds to a maximum dose of 25 mg applied in two weeks (1.8 mg HB/day), to avoid inhibition of the hypothalamic–pituitary–adrenal axis, and other systemic effects [17,19,20]. According to the literature [20], about 6% of the applied dose reaches systemic circulation. The formulation HB–menthone presented a permeated amount of approximately 40 μg/day from a 0.1% formulation with a surface area of 0.64 cm^2^, which is within the parameters established.

One limitation of this study is that ex vivo permeation tests are performed on healthy skin (due to confidentiality agreements, it is not possible to know if the donor had healthy or diseased skin beyond the TEWL test). In the future, research should test formulations on diseased human skin (psoriasis, dermatitis), considering the differences of permeation in diseased skin and the metabolic effects, if there are any. Thus, it would be possible to determine which formulation would be more suitable for each specific disease and patient, according to the disease characteristics and the patient’s conditions.

## 4. Materials and Methods

### 4.1. Materials

HB was purchased from Capot Chemical Company Limited (Hangzhou, China). The permeation enhancers carene ((+)-3-carene), cetiol (decyltetradecyl ethylhexanoate), decanol (decyl alcohol), limonene ((*S*)-4-isopropenyl-1-methyl cyclohexene), linoleic acid (9-*cis*,12-*cis*-linoleic acid), menthone ((2*S*,5*R*)-2-isopropyl-5-methylcyclohexanone) and nonane (*n*-nonane) were acquired from Sigma Aldrich (Madrid, Spain) and Azone^®^ (1-dodecylazacycloheptan-2-one) from Durham Pharmaceuicals (Durham, UK). Transcutol^®^ (diethylene glycol monoethyl ether) was kindly given by Gattefossé (Barcelona, Spain). MilliQ Plus system was used to obtain purified water. All the other chemical reagents used were of analytical grade.

### 4.2. Methods

#### 4.2.1. HPLC Validation Methodology

The HPLC equipment used for the analysis of these experiments was a Waters^®^ Alliance 2695 separation module with Kromasil^®^ C18 (5 μm, 15 × 0.46 mm) column (Technokroma, Barcelona, Spain). The mobile phase of acetonitrile/water (63:37) was under isocratic elution at a flow rate of 0.8 mL/min. A diode array detector Waters^®^ 2996 at a wavelength of 238 nm was used to detect HB. A volume of 50 μL of sample were injected. Data was processed using Empower 3^®^ Software.

For the preparation of stock solution, 1.25 mg of HB were weighed out and dissolved in 25 mL of Transcutol^®^. A mixture of Transcutol^®^/water (T/w) 70:30 (*v*/*v*) was used for the preparation of the standards at concentrations of 0.5–1.0–5.0–10.0–15–20–25 μL/mL. The same solvent mixture was used as a blank. Five independent calibration sets were prepared, each one on a different day.

The validation process of this methodology was performed according to the ICH guidelines [68] The parameters of specificity, accuracy, precision, linearity, range, detection limit, and quantification limit were analyzed.

Specificity is defined by the ICH guidelines as the capability to assess, without mistake, the analyte under evaluation in the presence of other expected components. Depending on the samples and their treatment, these components may include matrix components, impurities, and reagents or solvents used in the process as required by the method. Specificity was assessed by the absence of interferences at the same retention time at which the analyte appears.

Accuracy is the ability of an instrument to approach the real value in a measurement. This parameter was calculated as follows:

%RE= ((Co − Cn)/Cn)(1)

where Co corresponds to observed concentration; Cn corresponds to nominal concentration and %RE represents mean percentage deviation (% Relative Error). For the assessment of accuracy, five calibration curves were prepared on different days.

Precision is the ability of an instrument to replicate a measurement with minimal variation and is expressed as the mean coefficient of variation (%CV). For the determination of this parameter, five standard curves were prepared.

Linearity is when, in a given range, the result obtained is directly proportional to the amount of analyte present in the sample. Five calibration curves were evaluated for linearity, each one with seven concentration levels. This parameter was evaluated by the determination coefficient (*r*^2^) obtained from the analysis of least-squares linear regression of the calibration sets. The processing of the data was using MS excel software (Microsoft Corporation, Redmond, WA, USA). Linearity was also determined by one-way analysis of variance (ANOVA) test, comparing the concentration of a tested standard with the ratio of the areas by Graph Pad Prism^®^ (version 6.01, Graph Pad Prism software, Inc., La Jolla, CA, USA)

The range represents the difference between the maximum and minimum values, and uses the same units as the data. The range is obtained according to the linearity studies, and indicates the concentration at which it has been proved that the method has a suitable level of linearity, accuracy and precision.

To define sensitivity of an analytical method, it is necessary to determine the limit of detection (LOD) and the quantification limit (LOQ). The first of these is defined as the minimum amount of analyte that can be detected, but not necessarily accurately quantified. The second LOQ is the minimum concentration than can be accurately quantified with acceptable accuracy and precision. The acceptable limits for precision and accuracy for calibration standards were set at 20% CV and ±15% respectively. The determination of LOD and LOQ in accordance with the ICH guidelines were estimated based on a standard deviation of the slope and the response, as follows:

LOD = [(3.3 s)/ρ](2)

LOQ = [(10 s)/ρ](3)

where “s” represents standard deviation of the Y-intercept, and “ρ” represents the slope on the calibration curve.

#### 4.2.2. Ex Vivo Assays

HB is a lipophilic and highly insoluble in water corticosteroid [46,69]. In the light of this, the formulations were prepared diluting the drug in Transcutol^®^ (to meet the Sink conditions, Cs > 20 mg/mL) and 5% *v*/*v* of each promoter in order to obtain a final concentration of 1.0 mg/mL HB in each sample solution.

##### Skin Permeation Assay

Human skin from the abdominal region was obtained from a healthy woman during plastic surgery and was used as a permeation membrane (Barcelona-SCIAS Hospital, Barcelona, Spain). The volunteer gave written informed consent and the experimental protocol was approved by the Bioethics Committee of the Barcelona-SCIAS Hospital. For the drug permeation study, a dermatome (Model GA 630, Aesculap, Tuttlingen, Germany) was used to cut a piece of skin with a thickness of 0.4 mm [35,70]. With the stratum corneum facing the donor compartment, the skin was placed between the donor and receptor compartment in a Franz-type cell [71] (Hanson Research, Chatsworth, CA, USA; Crown Glass Company, INC, Jersey City, NJ, USA) with a 0.64 cm^2^ diffusion area, and fixed with paraffin film to prevent it from leaking. T/w (70:30) was used as a receptor solution in the receptor chamber. The cells were connected to a controlled bath at a temperature of 32 °C throughout the complete experiment. The skin was allowed to equilibrate for 30 min prior to the application of the formulation, and skin barrier integrity was assessed by measuring transepidermal water loss (TEWL) (TEWL-meter TM210, Courage & Khazaka, Koln, Germany). The probe was placed on the donor compartment in close contact with the skin, and lightly pressed to record the skin moisture content. Human skin pieces exhibiting TEWL values below 10 g/m^2^·h were used [72]. One milliliter of each formulation was placed onto the donor compartment in contact with the epidermal side of the skin. At a given time interval, 300 μL of sample from receptor compartment were withdrawn and replaced with the same volume of receptor solution, until there had been 24 h of contact. The amount of drug permeated was determined using HPLC and permeation parameters such as flux (J), permeability coefficient (Kp), amount permeated at 24 h (A_24_), and the drug amount retained (As) in skin was calculated using a linear least-squares regression model with GraphPad Prism^®^ (version 6.01, GraphPad Prism software, Inc.) software. All samples were made by using skin from the same donor, so as to diminish the variability of the response due to biological differences.

##### Amount of Drug Retained in Skin

Once the permeation study was finished, the skin was removed from the cell and used to determine the amount of drug retained using the protocol described elsewhere [73]. The skin was cleaned with a gauze soaked in solution of 0.05% of sodium dodecyl sulfate and distilled water to remove the excess of formulation on the surface [74]. The diffusional area of the skin in contact with the formulation was isolated and weighed. For the drug extraction, the skin was punctured with a needle, placed in a vial with 1 mL of acetonitrile and sonicated during 20 min at room temperature. The solutions obtained were measured directly by HPLC, indicating the amount of drug retained in the skin expressed in (μg·g^−1^ cm^−2^). Nonparametric Mann–Whitney statistical tests were performed to compare drug retention from different formulations [37].

##### Recovery Percentage

For this determination, a piece of skin of the same donor was weighed and placed in a tube with 1 mL of solution with a known concentration of HB in Transcutol^®^. The tube was kept in a bath for twenty-four hours at a temperature of 32 °C. After this time, the skin was removed, and the supernatant was measured by HPLC to determine the amount of drug that had penetrated. The amount of drug retained in the skin was determined using the same extraction procedure previously described for the samples. The recovery percentage was calculated as follows:

Recovery (%) = Amount retained/Amount penetrated × 100(4)

This recovery percentage was used to calculate and correct the amount of HB retained in samples.

#### 4.2.3. In Vivo Assays

Two in vivo tests were conducted in order to evaluate the tolerance and the efficacy of the drug. The Draize skin test was performed on male albino rabbits of 1.9–2.0 kg weight following the current international guidelines [35] to determine skin tolerance. Approval was obtained by the Animal Research Ethical Committee of the University of Barcelona, according to the regulations of the local government (Decree 214/1997, 30 July). Twenty-four hours before the test, the rabbit’s back was shaved with an electric razor, revealing two squares of 5 × 5 cm each, and scarred with a lancet. Then, 0.5 mL of HB–promoter solution was applied on each square, and the site was left uncovered until the next day. In accordance with the principles of 3R (reduction, refinement, and replacement) only three animals were used for each HB formulation [74]. After 24 h of exposure to the selected formulation, the excess was removed, and the skin was scored for edema and erythema (both graded from 0 to 4). The individual primary irritancy index was determined for each rabbit, adding the edema and erythema scores based on a standard scale. The mean value of the three rabbits’ scores was calculated. Taking into account the primary irritancy index value, they were classified as “nonirritant” (0–0.5), “mildly irritant” (0.5–2) “moderately irritant” (2–5), or “severely irritant” (5–8) [38].

The histamine-induced wheal suppression test was performed in order to compare the efficacy of the formulations with the best kinetic parameters [39]. In the procedure, New Zealand male albino rabbits of 1.9–2.0 kg were used. The animals were kept in standard cages with food and drink ad libitum. The day prior to the experiment, the rabbits’ back hair was shaved with an electric razor, avoiding harming their skin. The left side was used as a control and the right side as a treatment zone. 0.5 mL of the HB formulation (0.05%) was applied on the treatment side, and 0.5 mL of Transcutol^®^ without drug was applied on the control side. One hour later, the excess of formulation was removed with cotton wool and the histamine test was performed. A solution of histamine dihydrochloride (0.05 mL of 0.1% in distilled water) was injected intradermally with an insulin syringe. The size (cm^2^) of the bleb generated by the injection was measured with a caliper at 10, 20, and 30 min. Statistical analysis was made using GraphPad Prism^®^ (version 6.01 GraphPad Prism software, Inc.) software.

## 5. Conclusions

Based on the results obtained, it can be concluded that the presence of different permeation enhancers allows modulating permeation profiles, and thus, they affect the drug’s efficacy. We can highlight that for a formulation with HB, menthone and/or nonane are the most relevant permeation enhancers to increase the permeation and retention of the drug in the skin, respectively. Therefore, HB formulations containing menthone or nonane are suggested as prototypes for further clinical assays focused on the treatment of skin inflammatory diseases, such as, for instance, psoriasis and atopic dermatitis, among others. Due to the characteristics of the formulations, they could be used in pharmaceutical dosage forms such as roll-on or a flask with dosing cap.

## Figures and Tables

**Table 1:** Permeation enhancers and their chemical classification.

Chemical Class	Example(s)
Fatty acids	Oleic acid, Undecanoic acid, Linoleic acid
Alcohols and Alkanols	Octanol, Nonanol, Decanol
Terpenes	Menthol, Thymol, Limonene, Carene
Sulfoxides	Dimethyl sulfoxide, Dodecyl methyl sulfoxide
Surfactants	Sodium lauryl sulfate, Cetiol, Sorbitan mono-oleate
Polyols	Propylene glycol, Polyethylene glycol
Amides	*n*,*n*-Dimethyl-*m*-toluamide
Ureas	Urea
Lactam	Laurocapram (Azone^®^)
Sugars	Cyclodextrins

Modified from [28,29].

**Table 2:** Accuracy and precision inter-day data for HB standards solutions.

Standard Concentration (μg/mL)	Calculated Concentration (μg/mL)	%RE	%CV
0.50	0.46 ± 0.04	7.52	8.12
1.00	1.01 ± 0.09	−1.29	9.18
5.00	4.97 ± 0.06	0.55	1.11
10.00	10.00 ± 0.12	0.01	1.15
15.00	14.97 ± 0.16	0.18	1.06
20.00	20.15 ± 0.50	−0.73	2.48
25.00	24.90 ± 0.26	0.38	1.05

**Table 3:** HB standard curve and respective area response factor.

Concentration (μg/mL)	Ratio 1	Ratio 2	Ratio 3	Ratio 4	Ratio 5
0.5	120,731.26	102,721.10	121,211.86	146,101.52	121,400.90
1	127,756.00	107,598.30	118,281.10	124,086.20	149,836.40
5	117,370.32	111,500.02	115,936.50	124,473.88	118,584.80
10	116,073.00	111,987.70	116,643.40	123,272.40	119,428.40
15	115,639.87	112,150.33	115,545.20	122,871.87	119,042.87
20	120,423.60	117,231.60	115,496.90	117,671.60	118,850.15
25	115,293.64	112,280.36	114,267.60	122,551.44	118,734.48

**Table 4:** Skin permeation parameters of HB in the presence of tested promoters. Data are represented as median (min–max).

Permeation Enhancer	J (μg·h^−1^ cm^−2^) (min–max)	Kp (cm h^−1^)·10^5^ (min–max)	A_24_ (μg)	As (μg·g^−1^ cm^−2^) (min–max)
Nonane	0.141 ^b,c,d,e,f,g,h^	14.1 ^b,c,d,e,f,g,h^	2.74 ^b,c,d,e,f,g,h^	302.70 ^b,c,d,e,f,g,h^
	(0.138–0.167)	(13.8–16.7)	(2.57–3.82)	(280.04–315.76)
Menthone	2.588 ^a,c,d,e,f,g,h^	25.9 ^a,c,d,e,f,g,h^	35.47 ^a,c,d,e,f,g,h^	214.04 ^c,d,e,f,g,h^
	(2.476–2.734)	(248–273)	(31.84–9.27)	(203.06–226.87)
Limonene	0.073	7.29 × 10^−5^	1.49	62.62 ^d,e,f,g,h^
	(0.061–0.078)	(6.10–7.81)	(1.40–1.77)	(55.57–68.34)
Azone	0.052 ^f,g^	5.19 ^f,g^	1.2	71.17 ^e,g,h^
	(0.046–0.056)	(4.60–5.56)	(0.93–1.55)	(65.75–76.45)
Carene	0.060 ^f^	6.03 ^f^	1.28	41.39 ^f,g,h^
	(0.059–0.064)	(5.90–6.40)	(1.09–1.61)	(36.57–45.02)
Decanol	0.082	8.22	1.57	74.85 ^g,h^
	(0.077–0.087)	(7.70–8.70)	(1.17–1.78)	(69.86–78.85)
Linoleic acid	0.078	7.79	1.52	24.74
	(0.073–0.087)	(7.30–8.70)	(1.22–1.72)	(18.76–26.02)
Cetiol	0.071	7.06	1.6	22.04
	(0.063–0.075)	(6.30–7.50)	(1.31–1.73)	(18.56–24.98)

Letters represent statistical significate differences (*p* < 0.05) ^a^ Nonane; ^b^ Menthone; ^c^ Limonene; ^d^ Azone; ^e^ Carene; ^f^ Decanol; ^g^ Linoleic acid; ^h^ Cetiol. Permeation parameters: flux (J); permeability coefficient (Kp); amount permeated at 24 h (A_24_); amount of drug permeated (As).
